# Reducing glucoamylase usage for commercial-scale ethanol production from starch using glucoamylase expressing *Saccharomyces cerevisiae*

**DOI:** 10.1186/s40643-021-00375-5

**Published:** 2021-02-25

**Authors:** Xin Wang, Bei Liao, Zhijun Li, Guangxin Liu, Liuyang Diao, Fenghui Qian, Junjie Yang, Yu Jiang, Shumiao Zhao, Youguo Li, Sheng Yang

**Affiliations:** 1 College of Biological Engineering, Henan University of Technology, Zhengzhou, 450001 Henan China; 2 State Key Laboratory of Agricultural Microbiology, College of Life Science and Technology, Huazhong Agricultural University, Wuhan, 430070 Hubei China; 3 Angel Yeast Co., Ltd, Yichang, 443000 Hubei China; 4 Biosense Suzhou Limited, Suzhou, 215021 Jiangsu China; 5 Shanghai Research and Development Center of Industrial Biotechnology, Shanghai, 201201 China; 6 Key Laboratory of Synthetic Biology, CAS Center for Excellence in Molecular Plant Sciences, Chinese Academy of Sciences, 300 Fenglin Road, Shanghai, 200032 China; 7 Huzhou Center of Industrial Biotechnology, Shanghai Institutes for Biological Sciences, Huzhou, 313000 Zhejiang China

**Keywords:** Starch bioethanol production, Glucoamylase producing *Saccharomyces cerevisiae* strain, Raw corn starch fermentation, Raw cassava starch fermentation, Ethanol production optimization

## Abstract

The development of yeast that converts raw corn or cassava starch to ethanol without adding the exogenous α-amylase and/or glucoamylase would reduce the overall ethanol production cost. In this study, two copies of codon-optimized *Saccharomycopsis fibuligera* glucoamylase genes were integrated into the genome of the industrial *Saccharomyces cerevisiae* strain CCTCC M94055, and the resulting strain CIBTS1522 showed comparable basic growth characters with the parental strain. We systemically evaluated the fermentation performance of the CIBTS1522 strain using the raw corn or cassava starch at small and commercial-scale, and observed that a reduction of at least 40% of the dose of glucoamylase was possible when using the CIBTS1522 yeast under real ethanol production condition. Next, we measured the effect of the nitrogen source, the phosphorous source, metal ions, and industrial microbial enzymes on the strain’s cell wet weight and ethanol content, the nitrogen source and acid protease showed a positive effect on these parameters. Finally, orthogonal tests for some other factors including urea, acid protease, inoculum size, and glucoamylase addition were conducted to further optimize the ethanol production. Taken together, the CIBTS1522 strain was identified as an ideal candidate for the bioethanol industry and a better fermentation performance could be achieved by modifying the industrial culture media and condition.

**Supplementary Information:**

The online version contains supplementary material available at 10.1186/s40643-021-00375-5.

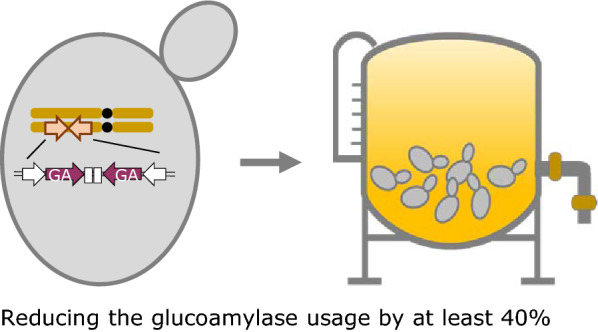

## Introduction

The conversion of starch into ethanol via hydrolysis–fermentation is practiced widely for the production of bioethanol, whiskey, beer, among others. The bioethanol was widely viewed as a potential new energy source and alternative to fossil fuels, Although, lignocellulosic material-based bioethanol has received more attention due to certain previously discussed advantages (Hahn-Hagerdal et al. 2006; Peplow 2014), it is still facing the unresolved challenges such as developing cost-effective lignocellulosic material pretreatment technologies, the robustness of the *Saccharomyces cerevisiae* strain, enabling the use of different sugars, and tolerating inhibitors present in the hydrolysate (Jansen et al. 2017). Thus, starch ethanol is still the dominant biofuel produced to date.

The conventional process for the fermentation of starch to ethanol is well established and mature technology that includes two main steps: (1) Starch is converted into glucose using α-amylase and glucoamylase. (2) Glucose is fermented by the *S. cerevisiae* to produce ethanol (Cripwell et al. 2020). In step one, the cost of the exogenous addition of the α-amylase and glucoamylase is estimated at US$0.048 per gallon of ethanol produced, which is equivalent to 8.3% of the total processing cost (Gorgens et al. 2015). Therefore, a genetically engineered amylolytic *S*. *cerevisiae* strain is strongly required to hydrolyze the starch and ferment the resulting sugars to ethanol (Chandel et al. 2018; Cripwell et al. 2019b).

The glucoamylase from different species have been cloned and expressed in *S. cerevisiae*. In 1985, Cetus Corporation firstly reported that *Aspergillus awamori* glucoamylase was successfully expressed in *S. cerevisiae* and the resulting strains were capable of growing on starch as the sole carbon source (Innis et al. 1985). *Aspergillus oryzae*, *Rhizopus oryzae*, *Saccharomyces diastaticus*, *Talaromyces emersonii*, and *S. fibuligera* glucoamylase genes were also expressed (Chi et al. 2009; Cripwell et al. 2019b; Favaro et al. 2012; Kotaka et al. 2008; Nakamura et al. 1997). To increase the level of the starch decomposition, α-amylase genes were also expressed in yeast combined with the glucoamylase, devoting to achieve liquefaction, hydrolysis and fermentation (consolidated bioprocessing, CBP) using a single organism (Altıntaş et al. 2003; Chen et al. 2008; Cripwell et al. 2019a, b; Kim et al. 2010; Liao et al. 2012; Nonato and Shishido 1988; Pretorius et al. 1991). However, the α-amylase expressing strain cannot produce sufficient starch degrading enzymes when inoculated, causing a longer fermentation time (~ > 120 h) at high starch loading situation (> 10%), (Gorgens et al. 2015; van Zyl et al. 2012). Therefore, a glucoamylase producing yeast strain in combination with α-amylase addition is a more practical approach.

Despite the fact that glucoamylase producing yeast strains have been widely developed, the researchers preferred to choosing the laboratory strains as the host because of the easier genetic manipulation (Gorgens et al. 2015). However, laboratory strains showed lower thermotolerance and decreased glucose fermentation rates when compared with industrial yeast strains (Kong et al. 2018). In addition, the starch-degrading enzymes genes were always expressed using episomal plasmids rather than integration into the genome, the selection marker and the copy number of the plasmids will affect the growth ability and cellular metabolism of the cell inevitably (Karim et al. 2013). More importantly, the fermentation performance tests of the constructed amylolytic yeast strains are usually done at a small-scale, which is far from the real bioethanol production environment (Gorgens et al. 2015). Meanwhile, there is a fundamental lack of understanding of how commonly used industrial nitrogen source, phosphorous source, metal ions, industrial enzymes, and culture conditions affect the amylolytic yeast strain’s ability to grow and produce ethanol.

For these reasons, we chose the widely used industrial bioethanol-producing *S*. *cerevisiae* strain CCTCC M94055 (hereafter referred to as AQ) as the host (Diao et al. 2013; Wang et al. 2019), integrated the codon-optimized *S. fibuligera* glucoamylase gene, which has been proven to have a high glucoamylase activity (Chi et al. 2009), into the δ sites on the genome. We firstly evaluated the basic growth performance of the newly constructed glucoamylase expressing strain compared with the parental AQ strain under different conditions. Next, we systemically evaluated the ethanol production capability of the glucoamylase expressing strain from corn or cassava starch at small and industrial scale. Finally, we studied how the nitrogen source, phosphorous source, metal ions, and the commonly used industrial enzymes, affect the strain’s ethanol production capability. An orthogonal test was also conducted to optimize ethanol production. As far as we know, this is the first study to thoroughly characterize this glucoamylase-producing yeast strain under real industrial condition and to provide a good reference to the bioethanol industry.

## Materials and methods

### Strains, plasmids, and growth conditions

Strains and plasmids used in this study are listed in Table 1. In brief, the industrial bioethanol production diploid *S. cerevisiae* strain AQ was used as the original host. All of the molecular cloning operations were conducted in *Escherichia coli* strain DH5α. Strain DH5α was cultured in LB medium at 37 ℃, and 100 μg/mL ampicillin was added if necessary. For routine *S. cerevisiae* molecular engineering, strains were maintained in YPD20 medium. For the induction of the *Cre* recombinase expression plasmid when deleting the G418 selection marker gene, strains were cultured in YPG20 medium. Antibiotics 300 μg/mL G418 or 400 μg/mL hygromycin was added. The composition of the medium used is shown in the Additional file 1: supporting information.

**Table 1:** Plasmids and strains used in this study

	Characters	Reference
Strains		
CCTCC M94055 (AQ)	Industrial ethanol producing *S. cerevisiae* strain, *MAT a/α*	Provided by Angel Yeast Co., Ltd
CCTCC M94055-GA	CCTCC M94055 δ::*P*_*ENO1*_-GA-*T*_*ENO1*_, *P*_*ADH1*_-GA-*T*_*PDC1*_	This study
CIBTS1518	CCTCC M94055-GA derivative	This study
CIBTS1519	CCTCC M94055-GA derivative	This study
CIBTS1520	CCTCC M94055-GA derivative	This study
CIBTS1521	CCTCC M94055-GA derivative	This study
CIBTS1522	CCTCC M94055-GA derivative	This study (CCTCC M2014657)
Plasmids		
pSH47-hph	*Cre* recombinase expression plasmid	Wang et al. (2019)
pYIE2-2GA-δ	Glucoamylase expression plasmid	This study
pYIE2-XKS1-PPP-δ	Backbone plasmid	Wang et al. (2019)

### Plasmids and strain construction

The KOD-plus-neo DNA polymerase (Toyobo, Japan) or KOD FX polymerase (Toyobo, Japan) was used for PCR amplifications. The DNA restriction enzymes for cloning were from Thermo Fisher Scientific (USA). The primers used for plasmid construction are all listed in Additional file 1: Table S1. The codon-optimized *S. fibuligera* glucoamylase gene (GenBank accession number: MW082635) was synthesized by GenScript Biotech Corporation (China). Detailed procedures for plasmid and strain construction are described in the Additional file 1: supporting information.

### Evaluation of the ethanol, glucose, NaCl, temperature and pH tolerance and ethanol production capability of the glucoamylase expressing strain

The 2°P wort (Provided by Angel Yeast Co., Ltd) containing various concentration of ethanol were used to determine the ethanol tolerance of the strains. The plate containing gradient concentration of glucose or NaCl were used to test the glucose and NaCl tolerance. The bubbles in the Duchenne tubule were used to evaluate the temperature and pH tolerance of the strains. The detailed procedures are described in the Additional file 1: supporting information.

### Fermentation test using corn and cassava starch at a small and industrial scale

The corn liquefied slurry used for the small-scale (350 mL) test was obtained from COFCO Biochemical Energy (Zhaodong) Co., Ltd. The reducing and total sugars contents were 5.9% and 26.5%, respectively. The yeast inoculum was prepared by mixing 10 g of active dry CIBTS1522 yeast with 143 mL of H_2_O and incubating at 35 ℃ for 20 min. Five milliliters of the strain culture were added into 350 mL of corn liquefied slurry (pH 4.6). 2.65 g of glucoamylase was dissolved in 100 mL of H_2_O and 5 mL (defined as a 100% dosage) were added to the fermentation broth. A volume of 2.5 and 1.5 mL of glucoamylase solution were added in parallel, which are 50% and 30% dosages, respectively. The fermentation was conducted at 32 ℃, 80 rpm for 72 h.

Industrial-scale bioethanol production from corn starch was conducted in Mengzhou Huaxing Alcohol Co., Ltd. The liquefaction and saccharification process was consistent with the existing one, except that glucoamylase was decreased to 0.22 kg/ton corn material, which is 70% of the previous dosage. Thirty kilograms of active dry CIBTS1522 strain were added to the 80 cubic meters seed tank, together with the saccharification mash. About 6 kg/ton corn material of urea was also added. After 3 h, another 40 cubic meters saccharification mash were added until the seed tank was full. After another 10–12 h, it was pumped to the fermentation tank to start the fermentation, the overall fermentation time was about 60 h.

To assess the ability of the recombinant strain to produce ethanol from cassava, the amylolytic CIBTS1522 strain was incubated in YPD20 medium for 24 h at 30 ℃, and inoculated into the YPCassava + Amylase + Glucoamylase, YPCassava + Amylase or YPCassava medium at 0.5 g/L inoculum size.

The 50 L scale bioethanol production from cassava starch was conducted in Guangxi COFCO Biomass Energy Co., Ltd. For this, 40 kg of cassava liquefied slurry were added to a 50 L fermentation tank, and 40 g of active dry CIBTS1522 yeast was inoculated. A 100% dosage of glucoamylase (1.15 kg/ton cassava starch) and urea (1.36 kg/ton cassava starch) were added. The fermentation was conducted at 30 ℃, 300 rpm, in aerobic condition in the first 4 h, and later in anaerobic condition, 33 ℃, 200 rpm.

### Optimization of the ethanol production by the glucoamylase expressing strain

To assess the effect of the different commonly used fermentation promoting factors on the bioethanol production by amylolytic strain CIBTS1522, 100 g of corn starch were placed into a 500 mL flask and about 240 mL of water were added. The starch slurry was adjusted to pH 6.0, heated above 90 °C to allow gelatinization and kept for 90 min in the presence of α-amylase (100 μL, 48,000 U/mL) to facilitate liquefaction. The mixture was cooled to 35 °C and adjusted to pH 4.5 with the addition of glucoamylase when necessary, various fermentation promotion factors were added according to the designed amount, and 0.2 g/100 g corn starch of dry CIBTS01522 strain were inoculated. The deionized water was added again to yield a raw corn starch to water ratio of 1:2.6, samples were taken at specific time intervals to determine the cell wet weight and ethanol content.

### Analytical methods

Cell densities (OD_600_) were measured using a Beckman Coulter DU 730 Spectrophotometer. The glucose, acetic acid, glycerol, and ethanol concentrations were detected using an Agilent 1200 HPLC, a Bio-Rad HPX-87H column and a refractive index detector. The column was eluted at 65 °C with 5 mM sulfuric acid at a flow rate of 0.6 mL/min.

In the industrial-scale fermentation test, the content of the total sugars and the reducing sugars were determined according to an existing protocol (http://egyankosh.ac.in/bitstream/123456789/12041/1/Experiment-4.pdf). The acidity was measured using the NaOH titration method. The alcohol was distilled, and the content was determined using alcohol meter. The number of the yeast cell was determined according to the report (Doran-Peterson et al. 2009).

## Results and discussion

### The industrial glucoamylase producing strain construction

**Fig. 1:**
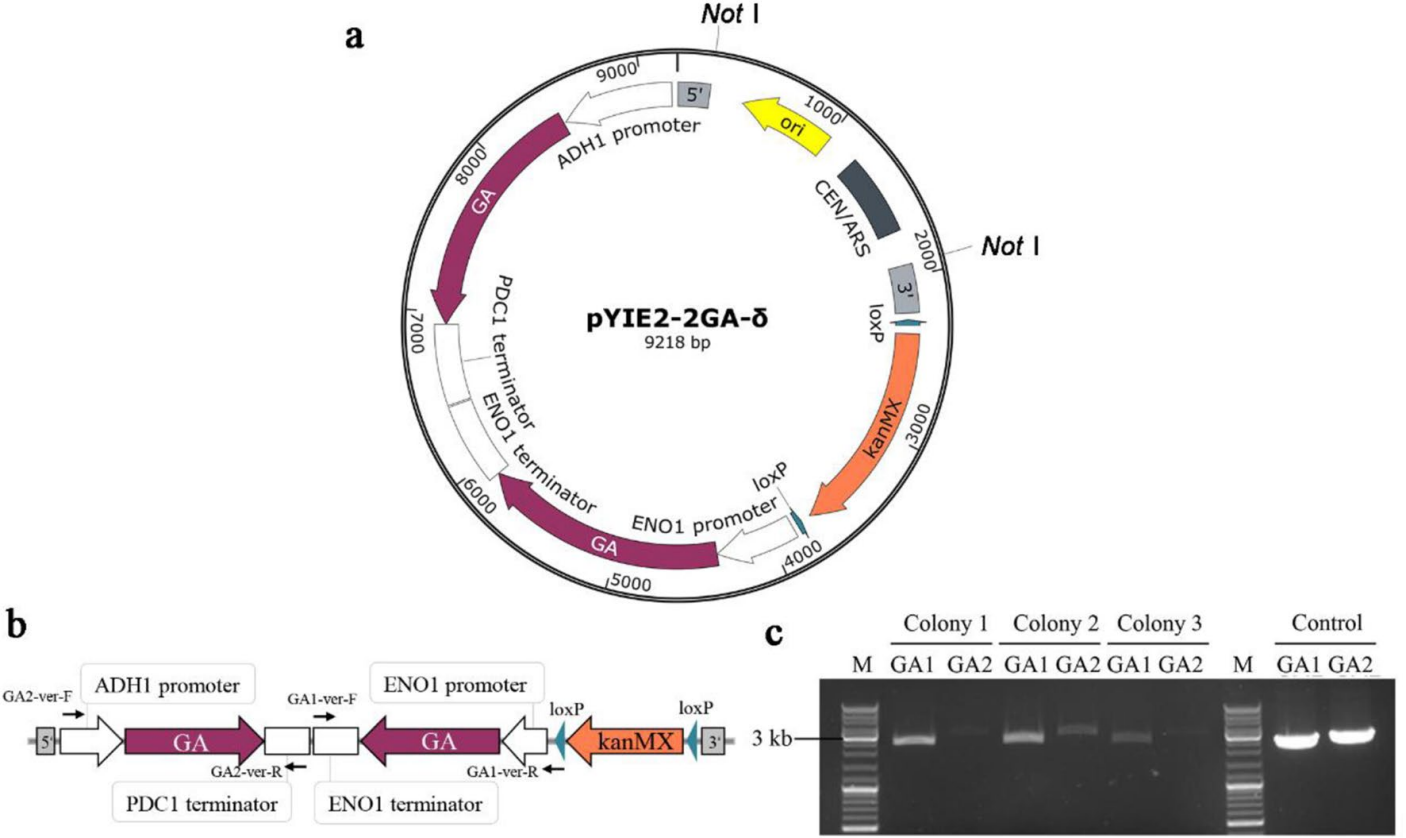
Construction of *S*. *fibuligera* derived glucoamylase expressing industrial *S. cerevisiae* strain. The map of the pYIE2-2GA-δ plasmid (**a**), the 5′ and 3′ were referred to as the up and down homology arm of the delta sequence, respectively. The *Not* I-linearized two copies of the GA expression cassette (**b**) and the agarose gel electrophoresis map of the verification of the GA integration, three transformants were picked randomly (**c**). GA1: *P*_*ENO1*_-GA-*T*_*ENO1*_ cassette. GA2: *P*_*ADH1*_-GA-*T*_*PDC1*_. Control: pYIE2-2GA-δ plasmid. M: DNA marker. GA: Glucoamylase

We chose the industrial Angel super dry yeast AQ as the original host, two copies of codon optimized *S*. *fibuligera* glucoamylase expression gene were cloned into plasmid pYIE2-2GA-δ (Fig. 1a) and expressed under the control of the strong promoter and terminator combinations *P*_*ENO1*_-GA-*T*_*ENO1*_ and *P*_*ADH1*_-GA-*T*_*PDH1*_, respectively. The two GA gene pairs were assembled in a tail-to-tail manner in case of the possible loss during the mitosis recombination (Fig. 1b). The *Not* I-linearized GA expression cassette was integrated into the δ sites of the AQ genome and verified using PCR using the primer pairs GA1-ver-F/GA1-ver-R and GA2-ver-F/GA2-ver-R (Fig. 1b), two copies of GA were integrated successfully (Fig. 1c). The *Cre* recombinase expression plasmid pSH47-hph was introduced to eliminate the G418 selection marker, later the pSH47-hph plasmid was cured and the resulting five colonies were named as CIBTS1518-CIBTS1522.

### The basic characteristics of the developed glucoamylase expressing *S. cerevisiae* strains

Table 2 illustrates the basic characters of the glucoamylase expressing yeast strains compared with the parental AQ yeast. In the presence of 12% ethanol, all strains showed obvious growth, and in the presence of 14% ethanol, only the parental strain, CIBTS1521 and CIBTS1522 showed little growth, and no growth was observed in the 16% ethanol conditions. Regarding glucose tolerance, all strains showed obvious growth using 50 g/L and 150 g/L glucose concentration conditions. At a glucose of 300 g/L condition, the control strain AQ showed moderate growth on day 1 and obvious growth at last, which was better than the glucoamylase expressing strains. Only CIBTS1522 and AQ strain were capable of growing at 400 g/L glucose, indicating their good potential use in high gravity fermentation. The performance pattern of the NaCl tolerance of strains was almost the same as that of glucose tolerance except the CIBTS1521 showed little growth on day 7 at 150 g/L NaCl.

**Table 2:** Physiological characteristics of the glucoamylase producing strains and the parental strain

		CCTCC M94055	CIBTS1518	CIBTS1519	CIBTS1520	CIBTS1521	CIBTS1522
Ethanol conc. (%, v/v)	12	+ + +	+ + +	+ + +	+ + +	+ + +	+ + +
	14	+	−	−	−	+	+
	16	−	−	−	−	−	−
Glucose conc. (g/L)^a^	5	+ + + / + + +	+ + + / + + +	+ + + / + + +	+ + + / + + +	+ + + / + + +	+ + + / + + +
	150	+ + + / + + +	+ + + / + + +	+ + + / + + +	+ + + / + + +	+ + + / + + +	+ + + / + + +
	300	+ + / + + +	+ + / + +	+ + / + +	+ + / + +	+ + / + +	+ + / + +
	400	+ / +	−/ + +	−/−	−/−	−/−	+ / +
NaCl conc (g/L)^a^	5	+ + + / + + +	+ + + / + + +	+ + + / + + +	+ + + / + + +	+ + + / + + +	+ + + / + + +
	50	+ / + +	+ / +	+ / +	+ / +	+ / + +	+ / +
	150	−/−	−/−	−/−	−/−	−/ +	−/−
	200	−/−	−/−	−/−	−/−	−/−	−/−
Temp. ℃^b^	57	+ + +					+ + +
	59	+ + +					+ + +
	61	+ + +					+ + +
	63	+					+ +
	65	−					−
pH^b^	2.0	−					−
	3.0	+ +					+ +
	4.0	+ + +					+ + +
	5.0	+ + +					+ + +
Ethanol titer from corn starch (%, v/v)^c^		21.6	22.5	22.1	22.1	21.7	22.6

+  +  + , obvious growth; +  + , moderate growth; + , little growth; −, no growth

^a^The results of days 1 and 7 are separated by the “/” symbol

^b^The temperature and pH tolerance test was only conducted using the control and CIBTS1522 strains

^c^The measurements represent the mean of three repeats

The CIBTS1522 strain exhibited better temperature tolerance than the control, even exposed to 63 ℃ for 10 min before culture. The CIBTS1522 strain showed moderate growth. The CIBTS1522 yeast could grow at pH 3.0 condition, which is consistent with AQ yeast results. Finally, we compared the ethanol titer from corn saccharified slurry of the preliminary strains, all the glucoamylase producing strains produced more ethanol than the control, which demonstrated the function of glucoamylase, especially the CIBTS1522, which produced 1 g/L more ethanol than the parental strain. Overall, the CIBTS1522 yeast exhibited excellent ethanol, glucose, NaCl, temperature, pH tolerance, and a higher ethanol production. Since these factors are significant physiological properties for high gravity fermentation process (Gibson et al. 2007), CIBTS1522 strain was chosen to perform the subsequent fermentation tests.

### Small and commercial-scale fermentation by CIBTS1522 using corn starch

**Fig. 2:**
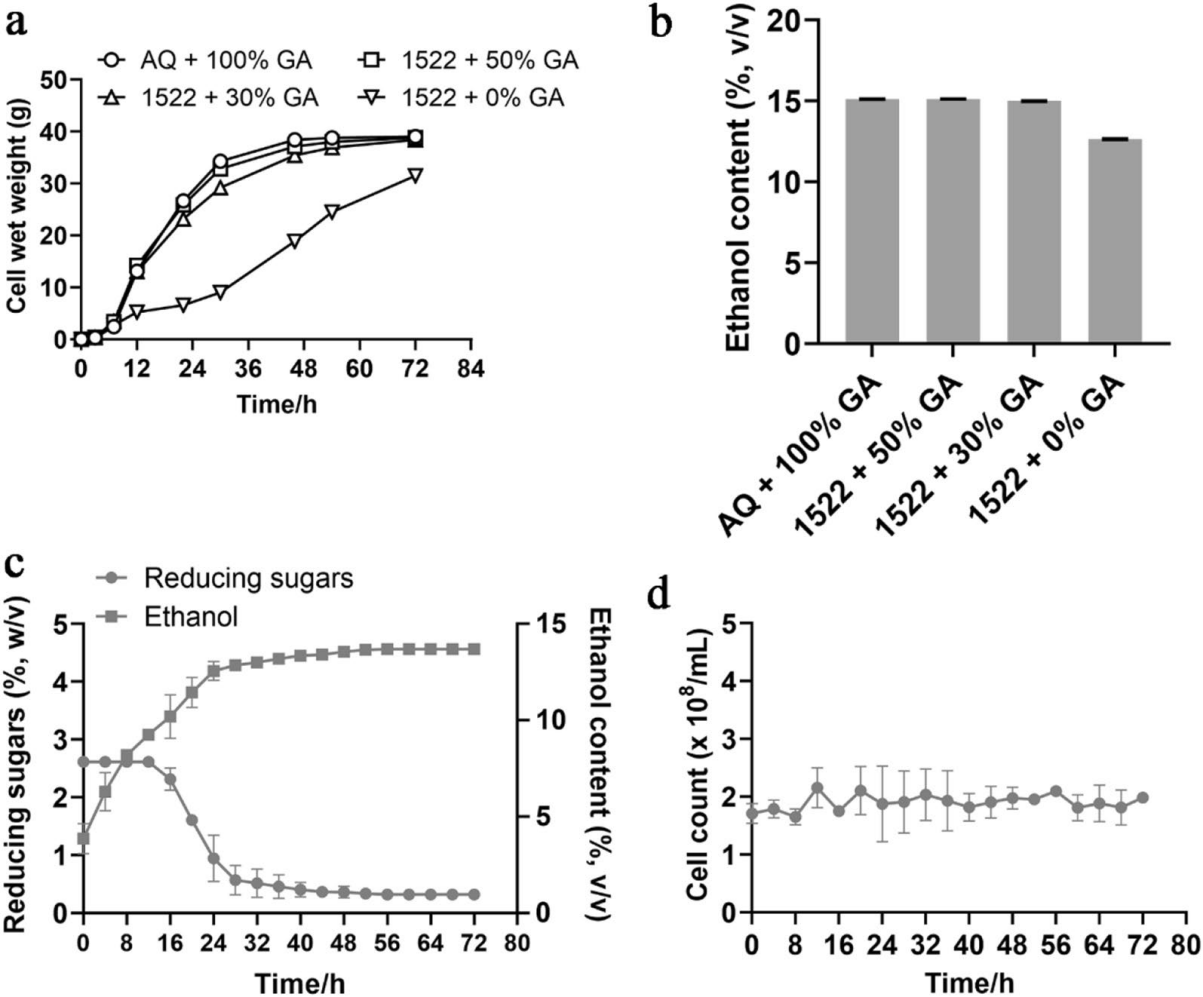
Corn starch fermentation by CIBTS1522 at a small (**a**, **b**) and industrial scale (**c**, **d**, respectively). The cell wet weight during the fermentation time (**a**) and the final ethanol content (**b**) at 72 h. 1522 was short for CIBTS1522, the control was AQ strain supplemented with 100% glucoamylase (**a**, **b**). At the industrial scale, the reducing sugars and ethanol content (**c**) and the cell count (**d**) were detected. Error bars represent the standard deviation from the mean of two replicates. *GA* glucoamylase

The cell wet weight, ethanol, and sugar production profiles during fermentation of corn starch liquefied slurry using AQ yeast at 100% glucoamylase loading and CIBTS1522 yeast with various glucoamylase loadings are illustrated in Fig. 2. In the small-scale conditions, the cell wet weight profiles of the respective enzyme supplementation condition were similar for the parental strain, except for CIBTS1522 without glucoamylase addition, which grew more slowly than the others (Fig. 2a). After 72 h, the CIBTS1522 supplemented with 50% and 30% glucoamylase loading produced 15.13% and 15.02% ethanol, respectively, which was similar to the control (15.12%), higher than the 0% glucoamylase condition (12.64%) (Fig. 2b; Table 3).

**Table 3:** Product formation by the CIBTS1522 strain after 72 h of fermentation in the corn starch liquefied slurry at small-scale

CIBTS1522	AQ + 100% GA	1522 + 50% GA	1522 + 30% GA	1522 + 0% GA
Final ethanol (%, v/v)	15.12	15.13	15.02	12.64
Glycerol (%, w/v)	1.18	1.09	1.03	0.80
Acetic acid (%, w/v)	0.06	0.04	0.04	0.03
Residual reducing sugars (%, w/v)	0.30	0.28	0.33	0.65
Ethanol productivity (g/L h)	2.10	2.10	2.09	1.76
Estimated ethanol yield (% of theoretical yield)^a^	85.28	85.34	84.72	71.30

The data represent the average of three independent tests

*GA* glucoamylase, *AQ* CCTCC M94055 strain

^a^34.25% (dw/v) raw starch loading is equivalent to 177.28 g/L ethanol (6.95 mol carbon), the theoretical ethanol yield from glucose is 0.51

Glycerol and acetic acid production are considered as an indicator of yeast stress, and typically about 1.2–1.5% glycerol concentrations are observed in starch ethanol production (Murthy et al. 2005). In our result, the glycerol and acetic acid were both maintained at lower levels (Table 3). The ethanol yield of 30% glucoamylase loading was comparable to that of the AQ yeast at 100% loading (Table 3). Maximum residual reducing sugars was observed at 0% glucoamylase loading (0.65%, w/v), indicating a relatively slow saccharification during fermentation. Taken together, we could save 70% of glucoamylase based on small-scale tests. The strain CIBTS1522 exhibited comparable fermentation capability to the previous reported strains (Additional file 1: Table S2).

More discreetly, we chose 70% glucoamylase loading under commercial-scale fermentation at Mengzhou Huaxing Alcohol Co., Ltd (China, Mengzhou). At 28 h, almost all of the sugars had been utilized and the ethanol achieved maximum of 13.7% (Fig. 2c), this was consistent with the parental strain supplemented with 100% glucoamylase loading (data not shown). The cell count was kept at approximately 2 × 10^8^/mL (Fig. 2d). The results demonstrated that at least 30% of glucoamylase could be saved without affecting ethanol productivity. It is estimated that the cost of glucoamylase is RMB 28/ton corn ethanol; when using the recombinant CIBTS1522 strain, the glucoamylase cost was decreased to RMB 19.6/ton corn ethanol. Assuming an annual production of 180,000 tons, the cost could be reduced by RMB 1.512 million per year.

### Small and large-scale fermentation using CIBTS1522 using cassava starch

**Fig. 3:**
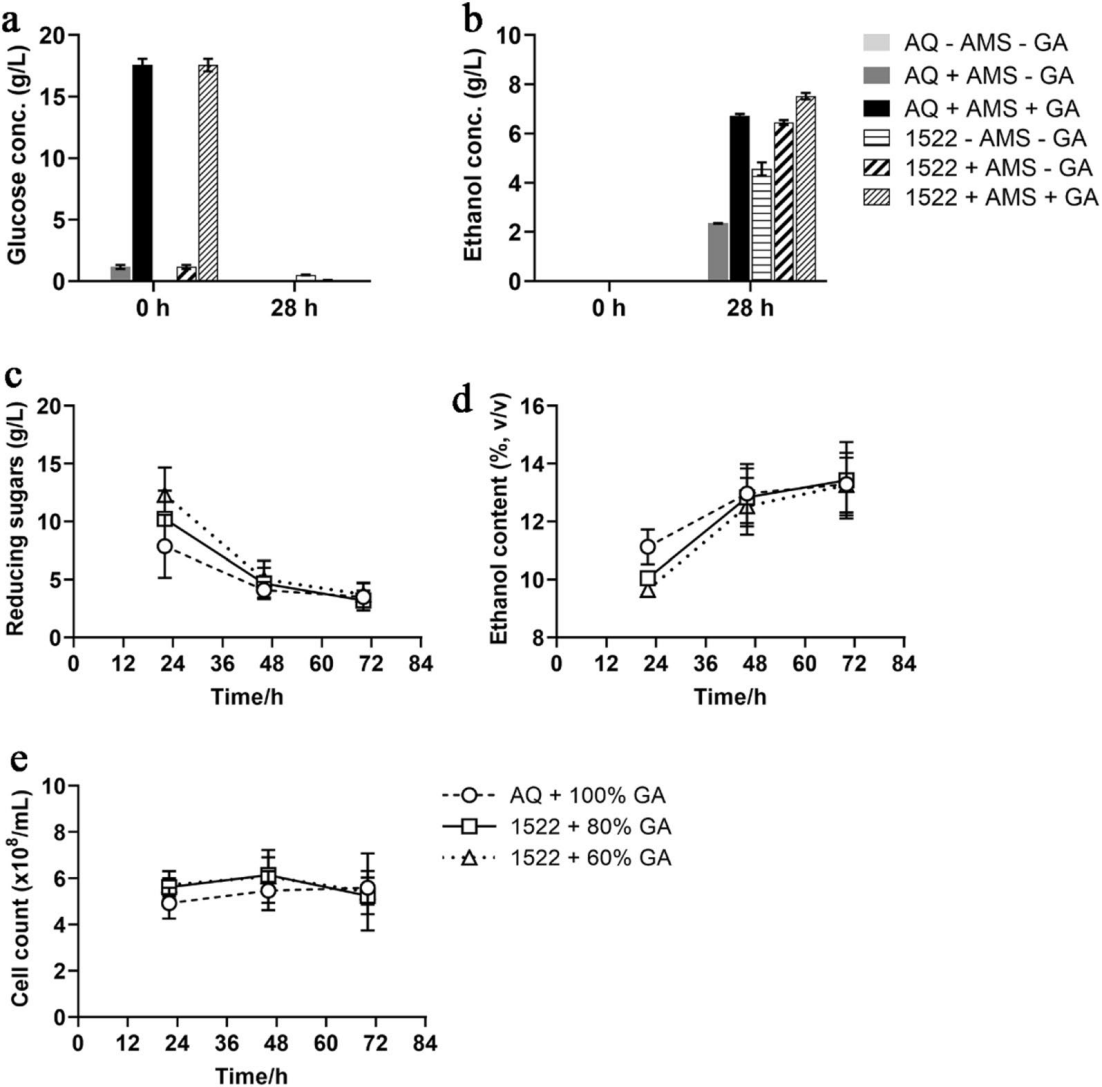
The fermentation performance of glucoamylase expressing yeast strains using cassava starch in small and large-scale fermentations. Glucose (**a**) and ethanol concentration (**b**) produced by the CIBTS1522 or the parental strain supplemented with or without α-amylase and glucoamylase under test tube conditions. **c**–**e** represent the reducing sugars (**c**), ethanol content (**d**), and cell count (**e**) under 50 L-scale fermentation condition by the parental and CIBTS1522 strains supplemented with different dosages of glucoamylase. *GA* glucoamylase, *AMS* α-amylase

To verify the wide application of the constructed glucoamylase expressing strain CIBTS1522, we chose cassava, another commonly used material when making bioethanol (Blagbrough et al. 2010), as the material to use for the small and large-scale bioethanol fermentation tests. When the cassava starch was pretreated with only α-amylase, few glucose (1.26 g/L) was produced, and when extra glucoamylase was added, 17.7 g/L of ethanol were produced (Fig. 3a). At 28 h, the CIBTS1522 strain supplemented without α-amylase and glucoamylase showed 0.52 g/L glucose residues, indicating that starch hydrolysis remains the rate limiting step in ethanol production. CIBTS1522 supplemented with only α-amylase produced an equivalent amount of ethanol compared with the AQ strain, when treated with both α-amylase and glucoamylase before fermentation, and a higher concentration of ethanol was produced by CIBTS1522 with the addition of glucoamylase (Fig. 3b).

**Fig. 4:**
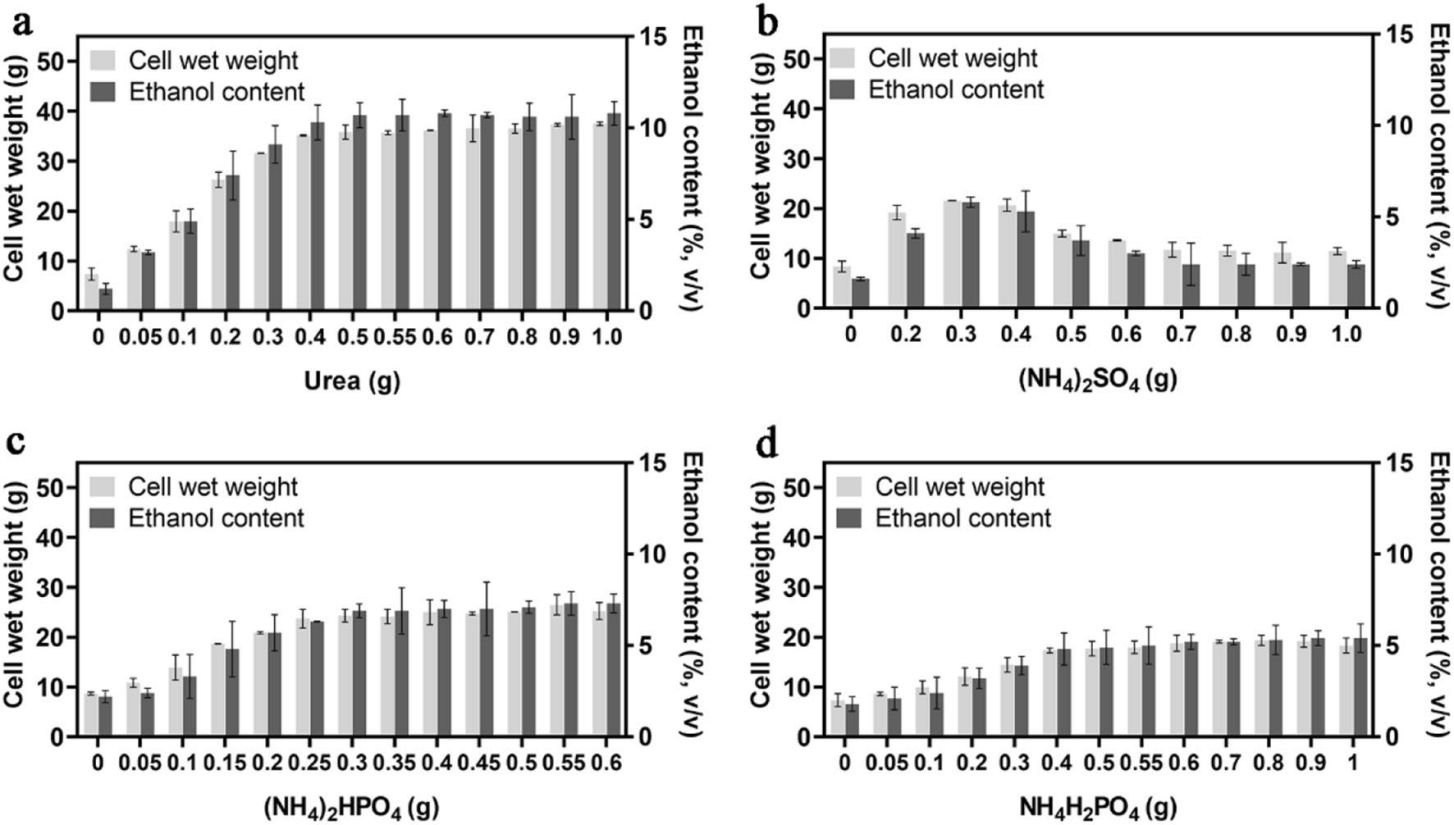
Effect of the nitrogen and phosphorous source on the cell wet weight and ethanol content of glucoamylase expressing strain CIBTS1522. The influence of the nitrogen source urea (**a**) and (NH_4_)_2_SO_4_ (**b**). The influence of the compound inorganic nitrogen and phosphorous sources (NH_4_)_2_HPO_4_ (**c**) and NH_4_H_2_PO_4_ (**d**) on these parameters. Error bars represent the standard deviation from the mean of three replicates

When the fermentation was scaled up to a 50 L bioreactor, at 22 h, the CIBTS1522 supplemented with 60% glucoamylase showed the highest reducing sugar concentration and the lowest ethanol production (Fig. 3 c and d). However, at 46 h, all the test strains showed the same residual reducing sugars concentrations and ethanol production, implying that the recombinant strain secreted the glucoamylase and matched the fermentation rate of the control over time. This was consistent with the corn starch fermentation results (Fig. 2a) and previous reports (Cripwell et al. 2019a). The cell count was stable during fermentation (Fig. 4e). Based on the fermentation results of cassava starch at a small and industrial-scale, we can conclude that at least 40% of glucoamylase could be saved when using the glucoamylase expressing strain CIBTS1522 (Fig. 3). The glucoamylase cost is about RMB 31.35/ton cassava ethanol, and the cost could be reduced by RMB 12.54/ton cassava ethanol when using the recombinant CIBTS1522 strain.

### Effect of the nitrogen and phosphorous source on the cell wet weight and ethanol content of CIBTS1522 cells

In commercial-scale bioethanol production, supplementation of the fermentation media with various nutrients, such as urea and ammonium sulfate, have been investigated for enhancing yeast cell performance and survival (Guillaume et al. 2019; Yue et al. 2012). We first evaluated the effect of nitrogen and phosphorous sources on the growth and ethanol production of the strain. As a commonly used nitrogen source, urea was contributed to the fermentation in the range of 0–0.4 g/100 g corn starch and no obvious promotion was observed when the amount increased further (Fig. 4a). When using the inorganic nitrogen source ammonium sulfate, the optimal concentration was 0.3 g/100 g corn starch (Fig. 4b), about 1 g/L, which was in agreement with the previous report (Duhan et al. 2013). For the compound nitrogen and phosphorous sources, (NH_4_)_2_HPO_4_ and NH_4_H_2_PO_4_, showed a similar pattern, but (NH_4_)_2_HPO_4_ had a better promoting function, with an optimal concentration > 0.3 g (Fig. 4c) and > 0.4 g (Fig. 4d), respectively. These results clearly demonstrated that the significant promotion function of the nitrogen and phosphorous sources.

### Effect of metal ions on the cell wet weight and ethanol content of CIBTS1522 cells

**Fig. 5:**
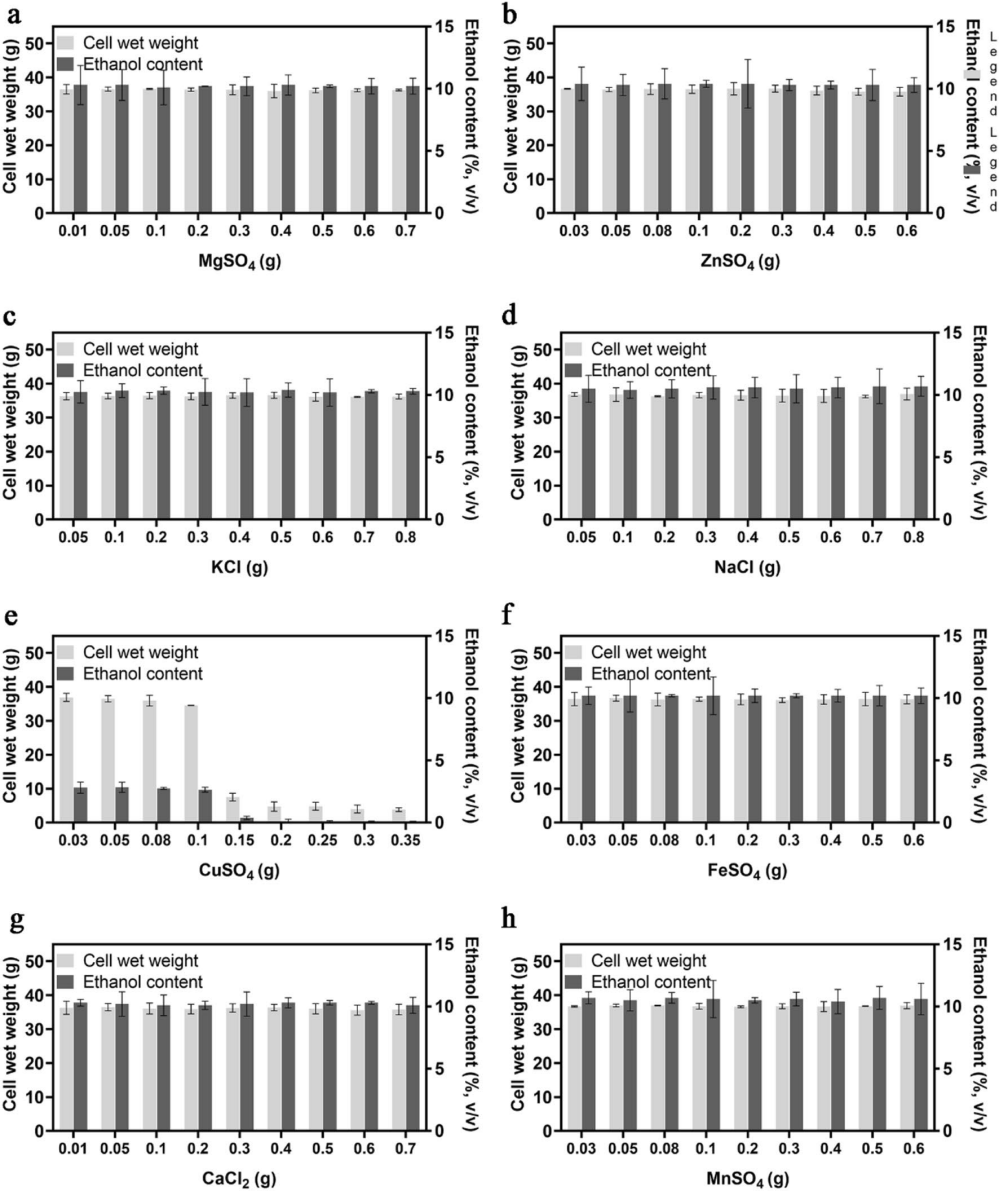
Effect of metal ions on the cell wet weight and ethanol content of glucoamylase expressing strain CIBTS1522. The influence of MgSO_4_ (**a**), ZnSO_4_ (**b**), KCl (**c**), NaCl (**d**), FeSO_4_ (**e**), CuSO_4_ (**f**), CaCl_2_ (**g**), and MnSO_4_ (**h**) were shown. Error bars represent the standard deviation from the mean of three replicates

Next we tested the impact of the metal ions on the fermentation of the strain. All of the tested metal ions showed no effect under the given concentration range, except for Cu^2+^ (Fig. 5). Metal ions such as Zn^2+^, Mg^2+^, and Mn^2+^ have been reported as the trace elements for yeast growth and ethanol fermentation (Walker 1998). Mg^2+^ and Zn^2+^ were proved to increase the heat and ethanol tolerance (Birch and Walker 2000; Zhao et al. 2009), while under normal condition and in the tested concentration range, Mg^2+^ and Zn^2+^ showed no influence on the growth and ethanol production of the strain (Fig. 5a, b). Previously reported that KCl and NaCl showed glucose utilizing inhibition above the concentration of 0.1 M (Casey et al. 2013). In our study, KCl and NaCl showed no effect on the fermentation of the strain at the given range of concentrations (Fig. 5c, d). Cu^2+^ showed a significant negative effect on the cell wet weight and ethanol production above 0.1 g (Fig. 5e). In addition, Fe^2+^, Ca^2+^, and Mn^2+^ showed no influence on the fermentation performance of the strain (Fig. 5f–h).

### Effect of microbial enzymes on the cell wet weight and ethanol content of CIBTS1522 and optimization of the ethanol production of the strain using an orthogonal test

**Fig. 6:**
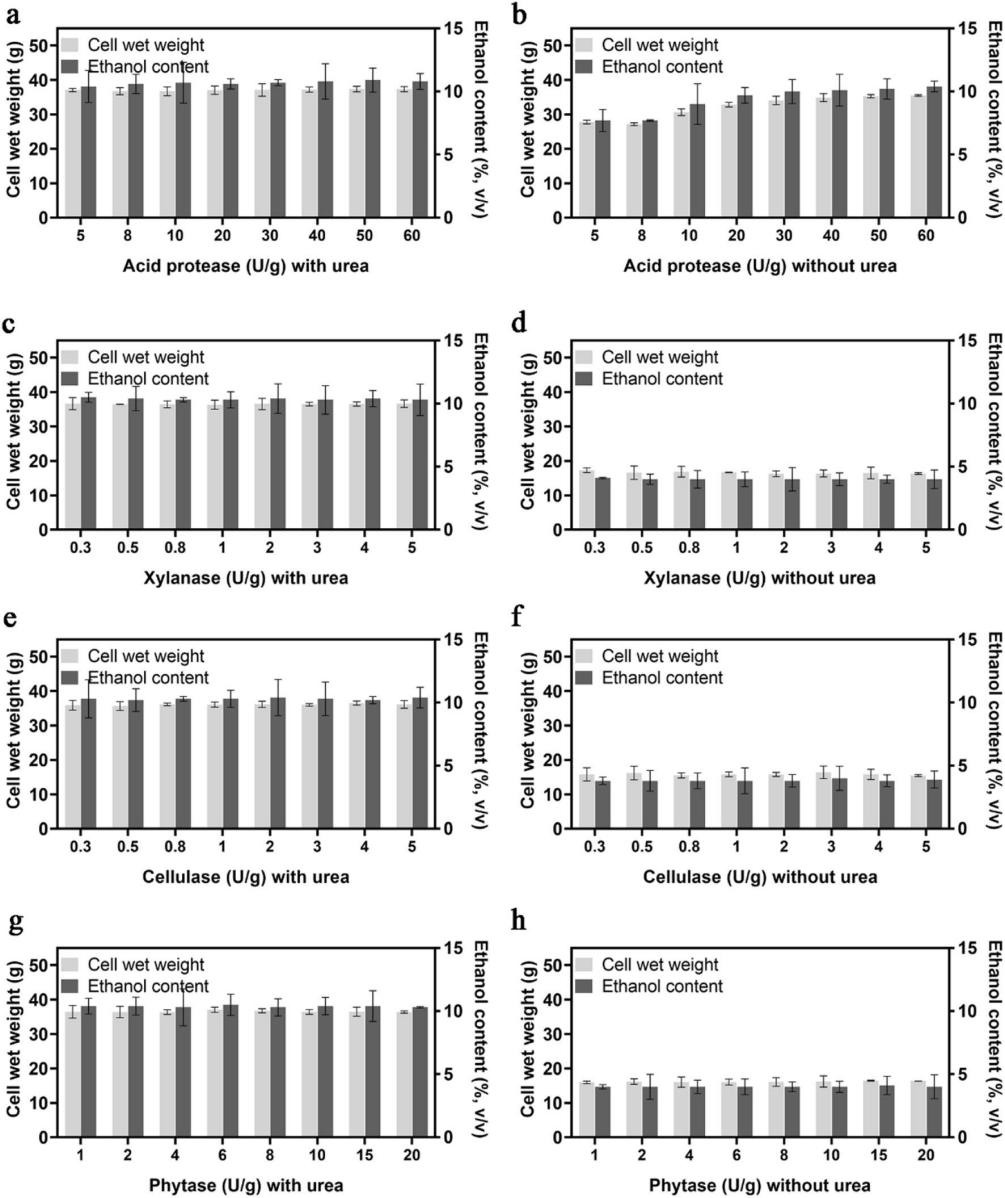
Effect of microbial enzymes on the cell wet weight and ethanol content of glucoamylase expressing strain CIBTS1522. The influence of the acid protease addition with (**a**) or without urea (**b**), the xylanase addition with (**c**) or without urea (**d**), the cellulase addition with (**e**) or without urea (**f**), and the phytase addition with (**g**) or without urea (**h**) was shown. Error bars represent the standard deviation from the mean of three replicates

The effects of four kinds of microbial enzymes (acid protease, xylanase, cellulase, and phytase) on the fermentation performance of the strain with or without the addition of urea were evaluated. The protease was proved to increase the fermentation rate (Vidal et al. 2009) and the ethanol yield by liberating free amino acids for the yeast (Johnston and McAloon 2014; Perez-Carrillo et al. 2012). Consistent with this, the cell wet weight and ethanol production increased with an increasing acid protease concentration from 0 to 20 U/g corn (Fig. 6b). When the urea was added, the function of the acid protease was masked (Fig. 6a).

When xylanase, cellulose, or phytase were added during the fermentation process, no positive effect was observed (Fig. 6c–h), which implied that the enzymes may function at other steps such as pretreatment procedure. For example, cellulases and xylanases may help in releasing the starch bound to the corn fiber, and induce cost and energy savings by decreasing the viscosity and reducing the binding of water to grains, thus facilitating the centrifugation and drying steps (Harris et al. 2014). Phytase has also shown positive impact on oil recovery in the corn dry grinding process (Luangthongkam et al. 2015). These results confirmed the distinct effect of urea on the fermentation improvement of the strain.

Based on the results of the single factor experiments conducted above, we chose urea and acid protease, together with the yeast inoculum size and exogenous glucoamylase, to design an orthogonal test to further optimize the fermentation conditions of the CIBTS1522 under high corn starch loading. The experimental design and result are shown in Table 4. The results showed that the order of the four factors regarding the ethanol content was: A > B = D > C, that is, the urea > acid protease = exogenous glucoamylase > inoculum size. The best combination should be A2B3C3D3; however, this combination was not included in the existing trial. We conducted an additional experiment to verify the hypothesis, indeed, the ethanol content of this combination was 14.0 ± 0.0 (%, v/v), corresponding to 96.4% of the theoretical ethanol yield, higher than the results among the trials. The results may be beneficial for further optimization of the industrial-scale ethanol production process.

**Table 4:** Orthogonal test design and results for the production of ethanol using CIBTS1522 under high ratio of raw corn starch to water (1:2.6)

Trial	A Urea (g/100 g corn starch)	B Acid protease (U/100 g corn starch)	C Yeast inoculum (g/100 g corn starch)	D Glucoamylase (U/100 g corn starch)	Ethanol content (%, v/v)
1	0.1	5	0.1	15	11.4
2	0.1	10	0.2	30	12.8
3	0.1	25	0.3	45	13.6
4	0.25	5	0.2	45	13.6
5	0.25	10	0.3	15	13.4
6	0.25	25	0.1	30	13.6
7	0.4	5	0.3	30	13.1
8	0.4	10	0.1	45	13.3
9	0.4	25	0.2	15	13.3
k1	12.600	12.700	12.767	12.700	
k2	13.533	13.167	13.233	13.167	
k3	13.233	13.500	13.367	13.500	
R	0.933	0.800	0.600	0.800	
Sum of squares of deviations	1.362	0.969	0.596	0.969	
Order	A > B = D > C				

## Conclusion

The glucoamylase expressing yeast strain CIBTS1522 was constructed and at least 30–40% of the dosage of glucoamylase could be reduced when fermenting the raw corn or cassava starch at an industrial-scale. We evaluated the effect of the nitrogen source, phosphorous source, compound inorganic nitrogen and phosphorous sources, metal ions, and industrial microbial enzymes on the cell wet weight and ethanol production of the strain. The nitrogen source and the acid protease showed significant positive effects on the fermentation performance of the strain. An orthogonal test including urea, acid protease, inoculum size, and glucoamylase addition was designed and conducted to further optimize ethanol production, and 14.0% ethanol could be produced, corresponding to 96.4% of the theoretical ethanol yield.

### Supplementary Information

**Additional file 1.** Supporting information.

## Data Availability

All data generated or analysed during this study are included in this published article (and its supplementary information files).

## Abbreviations

CBP: Consolidated bioprocessing
GA: Glucoamylase
AMS: α-Amylase
