# Supplementary material for: Reducing glucoamylase usage for commercial-scale ethanol production from starch using glucoamylase expressing Saccharomyces cerevisiae
Source: Bioresour Bioprocess. 2021 Feb 25;8(1):20. doi: 10.1186/s40643-021-00375-5 (PMC10992596; doi:10.1186/s40643-021-00375-5)
Supplement: Supplementary file 1 — Additional file 1. Supporting information. [file 40643_2021_375_MOESM1_ESM.docx]

Supporting information for Bioresources and Bioprocessing

**Reducing glucoamylase usage for commercial-scale ethanol production from starch using glucoamylase expressing *Saccharomyces cerevisiae***

Xin Wang^1#^, Bei Liao^2,3#^ Zhijun Li^3^, Guangxin Liu^3^, Liuyang Diao^4^, Fenghui Qian^5^, Junjie Yang^6^, Yu Jiang^7^, Shumiao Zhao^2^, Youguo Li^2^, Sheng Yang*^6^

^1^College of Biological Engineering, Henan University of Technology, Zhengzhou 450001, Henan, China

^2^State Key Laboratory of Agricultural Microbiology, College of Life Science and Technology, Huazhong Agricultural University, Wuhan 430070, Hubei, China

^3^Angel Yeast Co., Ltd, Yichang 443000, Hubei, China

^4^Biosense Suzhou Limited, Suzhou 215021, Jiangsu, China

^5^Shanghai Research and Development Center of Industrial Biotechnology, Shanghai 201201, China

^6^Key Laboratory of Synthetic Biology, CAS Center for Excellence in Molecular Plant Sciences, Chinese Academy of Sciences, Shanghai 200032, China
^7^Huzhou Center of Industrial Biotechnology, Shanghai Institutes for Biological Sciences, Huzhou 313000, Zhejiang, China

^#^These authors contributed equally to this work.

*Corresponding author: Sheng Yang ([syang@sibs.ac.cn](mailto:syang@sibs.ac.cn))

Mailing address: 300 Fenglin Road, CAS Center for Excellence in Molecular Plant Sciences, Chinese Academy of Sciences. Shanghai, China.

Tel: 86-21-54924173; Fax: 86-21-54924015.

**Medium**

LB medium: 5 g/L yeast extract, 10 g/L tryptone, 10 g/L NaCl, 15-20 g/L agar was added when solid plates were used.

YPD20 medium: 10 g/L yeast extract, 20 g/L tryptone, 20 g/L D-glucose,15-20 g/L agar was added when solid plates were used.

YPG20 medium: 10 g/L yeast extract, 20 g/L tryptone, 20 g/L galactose,15-20 g/L agar was added when solid plates were used.

YPCassava medium: 2% dw/v cassava starch, 10 g/L yeast extract, 20 g/L tryptone.

YPCassava + Amylase medium: 5 g of cassava sample was ground and dissolved in 100 mL H2O, α-amylase (0.4-0.6 g/kg raw cassava starch) was added and kept at 85℃-97℃ for 30 min, pH was adjusted to 5.4-5.8. Yeast extract and tryptone were added at the final concentration of 10 g/L and 20 g/L, respectively. Another waster was added until the final concentration of the cassava was 2% dw/v.

YPCassava + Amylase + Glucoamylase medium: To prepare the YPCassava+Amylase+Glucoamylase medium, glucoamylase (410-450 μL/kg raw cassava starch) was added after the liquefaction process, the other procedure was consistent with the YPCassava+Amylase medium.

Seed culture medium: 12 g/L glucose, 2 g/L KH_2_PO_4_, 0.5 g/L MgSO_4_, 1 g/L NaH_2_PO_4_, 1 g/L NH_4_NO_3_, 1 g/L trace elements containing 11 g/L ZnSO_4_, 1 g/L FeSO_4_, 6 g/L MnSO_4_, 0.3 g/L CoCl_2_, 40 g/L CuSO_4_, 0.6 g/L H_3_BO_3_, pH 5.8.

**Plasmids construction**

For the two copies of GA containing plasmid pYIE2-2GA-δ construction, the “δ-Ecori-Scori-δ-loxP-kanMX-loxP” fragment including the yeast CEN/ARS sequence and *E. coli* pUC replication origin, the G418 selection marker gene, the delta sequences for the homology recombination and the *Bam*H I and *Kpn* I restriction sites, were amplified from plasmid pYIE2-XKS1-PPP-δ using Primer 1 and Primer 2. Using the CCTCC M94055 genome as template, the *ENO1* promoter, *ENO1* terminator, *ADH1* promoter, *PDC1* terminator were amplified using the Primer 3/Primer 4 pairs, Primer 7/Primer 8 pairs, Primer 9/Primer 10 pairs and Primer 13/Primer 14 pairs, respectively. GA was amplified from the synthetic codon-optimized GA gene sequence using Primer 5/Primer 6 and Primer 11/Primer 12, respectively. The *ENO1* promoter, GA gene, *ENO1* terminator were fused by the overlap PCR flanked with the *Kpn* I and *Nde* I restriction sites. The *ADH1* promoter, GA sequence, *PDC1* terminator were also fused by the overlap PCR flanked with the *Nde* I and *Bam*H I restriction sites. The two GA expression cassettes and the backbone sequence were ligated to make the final plasmid pYIE2-2GA-δ.

**Strains construction**

For the CCTCC M94055-GA strain construction, the GA expression cassette was integrated at the δ site in strain CCTCC M94055-GA by transforming *Not* I-linearized pYIE2-2GA-δ plasmid using G418 as the selection marker, the yeast transformation was performed using standard LiAc transformation method. The successful integration colony was confirmed by diagnostic PCR using the primer pairs GA1-ver-F/ GA1-ver-R and GA2-ver-F/ GA2-ver-R. The *Cre* recombinase expression plasmid pSH47-hph was transformed into the CCTCC M94055-GA strain to eliminate the G418 selection marker, the resulting colony was verified on YPD20 agar plate with or without G418 antibiotic. The final strains were named CIBTS1518, CIBTS1519, CIBTS1520, CIBTS1521, CIBTS1522.

**Evaluation of the ethanol, glucose, NaCl, temperature and pH tolerance and ethanol production capability of the glucoamylase expressing strains**

To test the ethanol tolerance, 1.26, 1.40 and 1.68 mL of ethanol was added to 8.40, 8.60 and 8.32 mL 2 °P wort (Provided by Angel Yeast Co., Ltd) to make 12%, 14% and 16% ethanol wort medium. The strains were firstly cultured in seed culture medium, and then inoculated into ethanol wort culture using the inoculation loop, cultures were incubated at 28 ℃ for 1 week. The growth of the strains was evaluated based on the number of bubbles.

To test the glucose and NaCl tolerance, one inoculation loop of strains was streaked on the YPD agar plates containing gradient concentration of glucose (5, 150, 300, and 400 g/L) or NaCl (5, 50, 150 and 200 g/L), respectively. The plates were incubated at 28 ℃ for 1 week. After that, the growth of the strains was observed.

To test the temperature tolerance, the strain was inoculated into the seed culture and incubated at 28 ℃ for 24 h. Then, 100 μL of strain culture were transferred to a new 10 mL seed culture medium. Meanwhile, another 100 μL of strain culture were inoculated into the 10 mL 5 °P wort (the pH was adjusted to 2.0, 3.0, 4.0 and 5.0, respectively, provided by Angel Yeast Co., Ltd) to determine the pH tolerance. The Duchenne tubule was placed in the test tube to observe the strains’ growth. For temperature tolerance, once transferred, the test tubes were immediately incubated in a 57 ℃, 59 ℃, 61 ℃ or 63 ℃ water bath for 10 min. The strains were cultured at 28 ℃ for 5-7 days to observe the bubble in the Duchenne tubule.

To test the ethanol production capability of the strains, 50 g raw corn starch were placed in a 500 mL iodine flask, 175 mL of water were added, followed by α-amylase (100 U/g corn starch), the starch was heated to 75℃-80 ℃ and kept for 30 min. The mixture was cooled to 60 ℃ and the pH was adjusted to 4.5, and glucoamylase (200 U/g dry corn) was added and the mixture was incubated for 1 h. The seed culture of the strain was inoculated until the temperature dropped below 40 ℃, the fermentation was conducted at 40 ℃ for 65 h and the ethanol content was measured using densitometry after distillation. The results are shown as percentages by volume at 20 ℃ after calculation (according to the ethanol density conversion table).

**Table S1** Primers used in this study

| Primer name | Primer sequence (5′–3′) |
| --- | --- |
| Primer 1 | taGGATCCtgttggaatagaaatcaactatc |
| Primer 2 | gtaGGTACCggccactagtggatctgatatcac |
| Primer 3 | gtaGGTACCcttctaggcgggttatctactgatc |
| Primer 4 | aaaacggtcaatctgatcattttgatttagtgtttgtgtgttgat |
| Primer 5 | cacacaaacactaaatcaaaatgatcagattgaccgttttcttga |
| Primer 6 | agaaggcttaatcaaaagctttacaataattcgatcaacttgttt |
| Primer 7 | agttgatcgaattattgtaaagcttttgattaagccttctagtcc |
| Primer 8 | taGGATCCtacatCATATGgaaagaggtttagacattggctcttc |
| Primer 9 | taGGATCCcgatttttttctaaaccgtggaata |
| Primer 10 | aaaacggtcaatctgatcattgtatatgagatagttgattgtatg |
| Primer 11 | aatcaactatctcatatacaatgatcagattgaccgttttcttga |
| Primer 12 | ataattagagattaaatcgcttacaataattcgatcaacttgttt |
| Primer 13 | agttgatcgaattattgtaagcgatttaatctctaattattagtt |
| Primer 14 | catCATATGtttcaatcattggagcaatcattttac |
| GA1-ver-F | tatcagatccactagtggccggtac |
| GA1-ver-R | gattggaaagaggtttagacattggctc |
| GA2-ver-F | tttcaatcattggagcaatcattttac |
| GA2-ver-R | atccttttgttgtttccgggtgtac |

The restriction enzyme cutting sites are shown in capital letters

**Table S2** Engineering of industrial *S. cerevisiae* yeast for starch conversion

| Strain | Amylase origin | Fermentation condition | Ethanol Conc.(g/L) / Fermentation time | Ethanol yield (% theoretical) | Reference |
| --- | --- | --- | --- | --- | --- |
| M2n[TLG1-SFA1] | δ:: *Thermomyces lanuginosus* GA, *S. fibuligera* AMS | 1 L, batch, 200 g/L raw corn starch | 64 / 10 days | 55 | (Favaro et al. 2015) |
| ERT12 | δ:: *T. emersonii* AMS and codon-optimized GA | 100 mL, batch, 200 g/L raw corn starch | 89.3 / 192 h | 86 | (Cripwell et al. 2019) |
| M2n T1 | δ:: *T. emersonii* AMS and codon-optimized GA | 100 mL, batch, 200 g/L raw corn starch | 98.1 / 192 h | 94 | (Cripwell et al. 2019) |
| ATCC 9763/  YIpδAGSAδ | δ:: *A. awamori* GA, *Debaryomyces occidentalis* AMS | 100 mL, batch, 200 g/L soluble starch | 103 / 6 days | 73.5 | (Kim et al. 2011) |
| GRI-117UK /pUDGAA | Integration vector: *A. oryzae* GA | Laboratory scale, 50 g/L liquefied starch | 18.5 / 48 h | 64.9 | (Kotaka et al. 2008) |
| CIBTS1522 | δ:: *S. fibuligera* GA | 350 mL, batch, 342.5 g/L liquefied starch | 126.4 / 72 h^a^ | 71.3^a^ | This study |

AMS: α-amylase. GA: glucoamylase.

a: The data represents the 0% GA loading condition in Table 3

**References**

Cripwell RA, Rose SH, Favaro L, van Zyl WH (2019) Construction of industrial Saccharomyces cerevisiae strains for the efficient consolidated bioprocessing of raw starch. Biotechnol Biofuels 12:201 doi:10.1186/s13068-019-1541-5

Favaro L, Viktor MJ, Rose SH, Viljoen-Bloom M, van Zyl WH, Basaglia M, Cagnin L, Casella S (2015) Consolidated bioprocessing of starchy substrates into ethanol by industrial Saccharomyces cerevisiae strains secreting fungal amylases. Biotechnol Bioeng 112(9):1751-1760 doi:10.1002/bit.25591

Kim HR, Im YK, Ko HM, Chin JE, Kim IC, Lee HB, Bai S (2011) Raw starch fermentation to ethanol by an industrial distiller's yeast strain of Saccharomyces cerevisiae expressing glucoamylase and alpha-amylase genes. Biotechnol Lett 33(8):1643-8 doi:10.1007/s10529-011-0613-9

Kotaka A, Sahara H, Hata Y, Abe Y, Kondo A, Kato-Murai M, Kuroda K, Ueda M (2008) Efficient and direct fermentation of starch to ethanol by sake yeast strains displaying fungal glucoamylases. Biosci Biotechnol Biochem 72(5):1376-9 doi:10.1271/bbb.70825
